# 5-Methyl-2-phenyl-2*H*-pyrazol-3-ol

**DOI:** 10.1107/S1600536808028559

**Published:** 2008-09-13

**Authors:** Qiang Wang, Yi Zhang, Rong Wang, Yi-Lin Yang, Feng Zhi

**Affiliations:** a Department of Neurosurgery, Third Affiliated Hospital of Soochow University, Changzhou 213003, People’s Republic of China; b Laboratory of Neuronal Injury and Protection, Third Affiliated Hospital of Soochow University, Changzhou 213003, People’s Republic of China

## Abstract

The title compound, C_10_H_10_N_2_O, known as Edaravone (MCI-186), was crystallized from methanol. The two independent mol­ecules in the asymmetric unit are linked through an O—H⋯O hydrogen bond. One mol­ecule adopts a ketone form, while the other adopts an enol form. In the crystal structure, mol­ecules are linked through inter­molecular N—H⋯O hydrogen bonds, forming chains running along the *b* axis.

## Related literature

For background to the compound, see: Watanabe *et al.* (1994 ▶); The Edaravone Acute Infarction Study Group (2003 ▶). For bond-length data, see: Allen *et al.* (1987 ▶). ▶ 
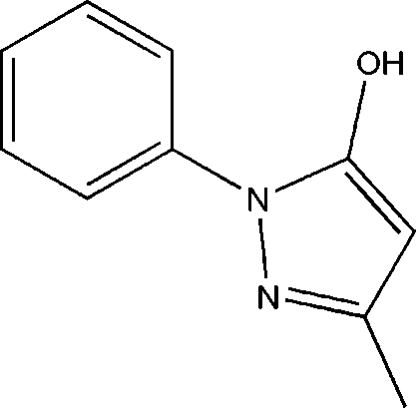


## Experimental

### 

#### Crystal data

C_10_H_10_N_2_O
*M* *_r_* = 174.20
Monoclinic, 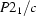

*a* = 10.336 (2) Å
*b* = 11.154 (2) Å
*c* = 15.863 (3) Å
β = 95.157 (3)°
*V* = 1821.4 (6) Å^3^
*Z* = 8
Mo *K*α radiation
μ = 0.09 mm^−1^
*T* = 298 (2) K
0.23 × 0.23 × 0.20 mm

#### Data collection

Bruker SMART CCD area-detector diffractometer
Absorption correction: multi-scan (*SADABS*; Sheldrick, 1996 ▶) *T*_min_ = 0.981, *T*_max_ = 0.983
10551 measured reflections
3923 independent reflections
2894 reflections with *I* > 2σ(*I*)
*R*_int_ = 0.025

#### Refinement

*R*[*F* ^2^ > 2σ(*F* ^2^)] = 0.040
*wR*(*F* ^2^) = 0.106
*S* = 1.04
3923 reflections
243 parameters
2 restraints
H atoms treated by a mixture of independent and constrained refinement
Δρ_max_ = 0.17 e Å^−3^
Δρ_min_ = −0.14 e Å^−3^

### 

Data collection: *SMART* (Bruker, 2002 ▶); cell refinement: *SAINT* (Bruker, 2002 ▶); data reduction: *SAINT*; program(s) used to solve structure: *SHELXS97* (Sheldrick, 2008 ▶); program(s) used to refine structure: *SHELXL97* (Sheldrick, 2008 ▶); molecular graphics: *SHELXTL* (Sheldrick, 2008 ▶); software used to prepare material for publication: *SHELXL97*.

## Supplementary Material

Crystal structure: contains datablocks global, I. DOI: 10.1107/S1600536808028559/sg2259sup1.cif

Structure factors: contains datablocks I. DOI: 10.1107/S1600536808028559/sg2259Isup2.hkl

Additional supplementary materials:  crystallographic information; 3D view; checkCIF report

## Figures and Tables

**Table 1:** Hydrogen-bond geometry (Å, °)

*D*—H⋯*A*	*D*—H	H⋯*A*	*D*⋯*A*	*D*—H⋯*A*
N2—H2*A*⋯N4^i^	0.907 (9)	1.904 (10)	2.7999 (17)	169.4 (17)
O2—H2*B*⋯O1	0.870 (9)	1.618 (10)	2.4813 (15)	170.9 (19)

Symmetry code: (i) 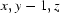.

**Fig. 1.**
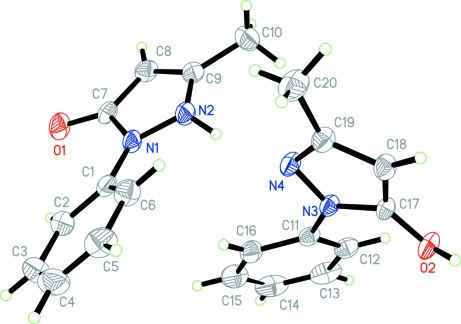
The structure of (I) at the 30% probability level. Intramolecular hydrogen bonds are shown as dashed lines.

**Fig. 2.**
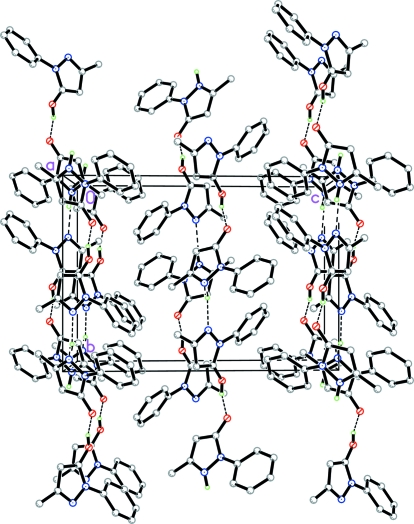
Molecular packing of (I), viewed along the a axis. Intermolecular hydrogen bonds are shown as dashed lines.

## supplementary crystallographic information

### Comment

Edaravone (5-methyl-2-phenyl-2*H*-pyrazol-3-ol, MCI-186) is a free-radical scavenger that was approved by the Ministry of Health, Labor, and Welfare of Japan in 2001, and is now widely used for the treatment of acute cerebral infarction (Watanabe *et al.*., 1994; The Edaravone Acute Infarction Study Group, 2003). We report in this paper the crystal structure of the compound, (I).

The compound consists of two independent molecules (Fig. 1), which are linked through an intramolecular O2—H2B···O1 hydrogen bond (Table 1). One molecule adopts a ketone form, while the other adopts an enol form. In the ketone molecule, the dihedral angle between the C1–C6 benzene ring and the N1/N2/C9/C8/C7 ring is 22.9 (2)°. In the enol molecule, the dihedral angle between the C11–C16 benzene ring and the N3/N4/C19/C18/C17 ring is 34.3 (2)°. In the compound, all the bond lengths are within normal ranges (Allen *et al.*, 1987). The bond length of C17—O2 [1.325 (2) Å] is longer than that of C7—O1 [1.260 (2) Å], which is caused by the enol form.

In the crystal structure, molecules are linked through intermolecular N–H···O hydrogen bonds (Table 1), forming chains running along the *b* axis (Fig. 2).

### Experimental

The crystal of the compound was recrystallized by edaravone in methanol.

### Refinement

H2A and H2B were located in a difference Fourier map and refined isotropically, with N–H and O–H distances restrained to 0.90 (1) and 0.85 (1) Å, and with *U*_iso_(H) set to 0.08 Å^2^. Other H atoms were constrained to idealized geometries, with C–H = 0.93–0.96 Å, and with *U*_iso_(H) set to 1.2*U*_eq_(C) and 1.5*U*_eq_(methyl C).

### Crystal data

**Table:**

C_10_H_10_N_2_O	*F*(000) = 736
*M**_r_* = 174.20	*D*_x_ = 1.271 Mg m^−^^3^
Monoclinic, *P*2_1_/*c*	Mo *K*α radiation, λ = 0.71073 Å
Hall symbol: -P 2ybc	Cell parameters from 3931 reflections
*a* = 10.336 (2) Å	θ = 2.3–29.0°
*b* = 11.154 (2) Å	µ = 0.09 mm^−^^1^
*c* = 15.863 (3) Å	*T* = 298 K
β = 95.157 (3)°	Block, colorless
*V* = 1821.4 (6) Å^3^	0.23 × 0.23 × 0.20 mm
*Z* = 8	

### Data collection

**Table:**

Bruker SMART CCD area-detector diffractometer	3923 independent reflections
Radiation source: fine-focus sealed tube	2894 reflections with *I* > 2σ(*I*)
graphite	*R*_int_ = 0.025
ω scan	θ_max_ = 27.0°, θ_min_ = 2.2°
Absorption correction: multi-scan (SADABS; Sheldrick, 1996)	*h* = −13→11
*T*_min_ = 0.981, *T*_max_ = 0.983	*k* = −13→14
10551 measured reflections	*l* = −20→18

### Refinement

**Table:**

Refinement on *F*^2^	Secondary atom site location: difference Fourier map
Least-squares matrix: full	Hydrogen site location: inferred from neighbouring sites
*R*[*F*^2^ > 2σ(*F*^2^)] = 0.040	H atoms treated by a mixture of independent and constrained refinement
*wR*(*F*^2^) = 0.106	*w* = 1/[σ^2^(*F*_o_^2^) + (0.0428*P*)^2^ + 0.3053*P*] where *P* = (*F*_o_^2^ + 2*F*_c_^2^)/3
*S* = 1.04	(Δ/σ)_max_ < 0.001
3923 reflections	Δρ_max_ = 0.17 e Å^−^^3^
243 parameters	Δρ_min_ = −0.14 e Å^−^^3^
2 restraints	Extinction correction: SHELXL, Fc^*^=kFc[1+0.001xFc^2^λ^3^/sin(2θ)]^-1/4^
Primary atom site location: structure-invariant direct methods	Extinction coefficient: 0.0087 (12)

### Special details

**Table:**

Geometry. All e.s.d.'s (except the e.s.d. in the dihedral angle between two l.s. planes) are estimated using the full covariance matrix. The cell e.s.d.'s are taken into account individually in the estimation of e.s.d.'s in distances, angles and torsion angles; correlations between e.s.d.'s in cell parameters are only used when they are defined by crystal symmetry. An approximate (isotropic) treatment of cell e.s.d.'s is used for estimating e.s.d.'s involving l.s. planes.
Refinement. Refinement of *F*^2^ against ALL reflections. The weighted *R*-factor *wR* and goodness of fit *S* are based on *F*^2^, conventional *R*-factors *R* are based on *F*, with *F* set to zero for negative *F*^2^. The threshold expression of *F*^2^ > σ(*F*^2^) is used only for calculating *R*-factors(gt) *etc*. and is not relevant to the choice of reflections for refinement. *R*-factors based on *F*^2^ are statistically about twice as large as those based on *F*, and *R*- factors based on ALL data will be even larger.

### Fractional atomic coordinates and isotropic or equivalent isotropic displacement parameters (Å^2^)

**Table:**

	*x*	*y*	*z*	*U*_iso_*/*U*_eq_	
O1	0.76081 (11)	0.23408 (9)	0.09473 (8)	0.0652 (3)	
O2	0.63865 (11)	0.42520 (9)	0.07652 (7)	0.0577 (3)	
N1	0.80235 (11)	0.03414 (9)	0.07061 (8)	0.0469 (3)	
N2	0.76341 (13)	−0.04917 (10)	0.00921 (8)	0.0516 (3)	
N3	0.69321 (11)	0.62596 (9)	0.06745 (7)	0.0418 (3)	
N4	0.76998 (12)	0.70070 (10)	0.02382 (8)	0.0490 (3)	
C1	0.91015 (13)	0.01075 (12)	0.12978 (9)	0.0457 (3)	
C2	0.92942 (16)	0.07840 (15)	0.20276 (10)	0.0603 (4)	
H2	0.8722	0.1399	0.2131	0.072*	
C3	1.03427 (19)	0.05375 (19)	0.26009 (12)	0.0768 (5)	
H3	1.0481	0.0997	0.3090	0.092*	
C4	1.1181 (2)	−0.0372 (2)	0.24600 (13)	0.0814 (6)	
H4	1.1881	−0.0536	0.2853	0.098*	
C5	1.09872 (17)	−0.10395 (18)	0.17403 (14)	0.0755 (5)	
H5	1.1557	−0.1661	0.1647	0.091*	
C6	0.99550 (15)	−0.08041 (15)	0.11475 (11)	0.0597 (4)	
H6	0.9837	−0.1255	0.0653	0.072*	
C7	0.73814 (14)	0.14046 (12)	0.05144 (10)	0.0486 (4)	
C8	0.65398 (16)	0.11675 (14)	−0.02083 (10)	0.0566 (4)	
H8	0.5970	0.1713	−0.0486	0.068*	
C9	0.66976 (15)	0.00076 (14)	−0.04331 (10)	0.0536 (4)	
C10	0.6047 (2)	−0.07170 (17)	−0.11335 (11)	0.0760 (5)	
H10A	0.6586	−0.0744	−0.1596	0.114*	
H10B	0.5227	−0.0359	−0.1321	0.114*	
H10C	0.5908	−0.1517	−0.0937	0.114*	
C11	0.61818 (14)	0.67394 (12)	0.12999 (8)	0.0441 (3)	
C12	0.49765 (15)	0.62664 (14)	0.14196 (10)	0.0545 (4)	
H12	0.4655	0.5611	0.1104	0.065*	
C13	0.42556 (18)	0.67832 (19)	0.20167 (12)	0.0755 (6)	
H13	0.3450	0.6462	0.2108	0.091*	
C14	0.4708 (2)	0.7760 (2)	0.24752 (12)	0.0860 (7)	
H14	0.4205	0.8110	0.2866	0.103*	
C15	0.5904 (2)	0.82150 (17)	0.23553 (11)	0.0799 (6)	
H15	0.6213	0.8876	0.2669	0.096*	
C16	0.66614 (18)	0.77067 (13)	0.17737 (10)	0.0584 (4)	
H16	0.7482	0.8012	0.1703	0.070*	
C17	0.70530 (13)	0.51085 (11)	0.04194 (9)	0.0430 (3)	
C18	0.79096 (15)	0.51055 (13)	−0.01882 (10)	0.0526 (4)	
H18	0.8190	0.4445	−0.0479	0.063*	
C19	0.82756 (15)	0.62954 (13)	−0.02813 (10)	0.0510 (4)	
C20	0.91583 (19)	0.68181 (17)	−0.08846 (13)	0.0794 (6)	
H20A	0.9060	0.7674	−0.0900	0.119*	
H20B	0.8938	0.6496	−0.1440	0.119*	
H20C	1.0042	0.6619	−0.0700	0.119*	
H2A	0.7692 (17)	−0.1287 (9)	0.0208 (11)	0.080*	
H2B	0.6823 (16)	0.3585 (12)	0.0773 (12)	0.080*	

### Atomic displacement parameters (Å^2^)

**Table:**

	*U*^11^	*U*^22^	*U*^33^	*U*^12^	*U*^13^	*U*^23^
O1	0.0757 (7)	0.0322 (5)	0.0864 (8)	0.0043 (5)	−0.0001 (6)	−0.0043 (5)
O2	0.0673 (7)	0.0311 (5)	0.0772 (7)	0.0007 (5)	0.0205 (6)	0.0059 (5)
N1	0.0531 (7)	0.0283 (6)	0.0577 (7)	0.0011 (5)	−0.0031 (6)	0.0011 (5)
N2	0.0640 (8)	0.0310 (6)	0.0581 (7)	0.0018 (5)	−0.0030 (6)	0.0012 (5)
N3	0.0493 (6)	0.0298 (6)	0.0476 (6)	0.0024 (5)	0.0114 (5)	0.0017 (5)
N4	0.0588 (8)	0.0324 (6)	0.0582 (7)	−0.0021 (5)	0.0185 (6)	0.0015 (5)
C1	0.0456 (8)	0.0362 (7)	0.0551 (8)	−0.0040 (6)	0.0031 (6)	0.0098 (6)
C2	0.0646 (10)	0.0555 (10)	0.0603 (10)	−0.0023 (8)	0.0035 (8)	0.0003 (8)
C3	0.0793 (13)	0.0876 (14)	0.0607 (11)	−0.0113 (11)	−0.0084 (9)	0.0024 (10)
C4	0.0653 (12)	0.1020 (16)	0.0736 (12)	−0.0004 (11)	−0.0122 (10)	0.0206 (12)
C5	0.0569 (10)	0.0769 (12)	0.0919 (14)	0.0161 (9)	0.0016 (10)	0.0187 (11)
C6	0.0567 (9)	0.0531 (9)	0.0687 (10)	0.0082 (7)	0.0024 (8)	0.0050 (8)
C7	0.0513 (8)	0.0310 (7)	0.0640 (9)	0.0023 (6)	0.0082 (7)	0.0075 (7)
C8	0.0597 (9)	0.0453 (9)	0.0635 (10)	0.0101 (7)	−0.0019 (8)	0.0127 (7)
C9	0.0599 (9)	0.0477 (9)	0.0523 (8)	0.0013 (7)	−0.0006 (7)	0.0069 (7)
C10	0.0886 (13)	0.0739 (12)	0.0622 (10)	−0.0045 (10)	−0.0120 (9)	−0.0032 (9)
C11	0.0544 (8)	0.0339 (7)	0.0447 (7)	0.0122 (6)	0.0093 (6)	0.0067 (6)
C12	0.0524 (9)	0.0531 (9)	0.0586 (9)	0.0130 (7)	0.0090 (7)	0.0135 (7)
C13	0.0686 (11)	0.0872 (14)	0.0749 (12)	0.0326 (10)	0.0304 (9)	0.0286 (11)
C14	0.1171 (18)	0.0853 (15)	0.0612 (11)	0.0533 (13)	0.0383 (12)	0.0143 (11)
C15	0.1298 (18)	0.0540 (11)	0.0579 (10)	0.0258 (11)	0.0198 (11)	−0.0063 (8)
C16	0.0812 (11)	0.0414 (8)	0.0537 (9)	0.0066 (8)	0.0121 (8)	−0.0019 (7)
C17	0.0495 (8)	0.0285 (6)	0.0511 (7)	0.0027 (6)	0.0048 (6)	0.0015 (6)
C18	0.0622 (9)	0.0379 (8)	0.0597 (9)	0.0065 (7)	0.0166 (7)	−0.0068 (7)
C19	0.0538 (8)	0.0432 (8)	0.0580 (9)	0.0022 (6)	0.0163 (7)	−0.0002 (7)
C20	0.0868 (13)	0.0683 (12)	0.0901 (13)	−0.0032 (10)	0.0467 (11)	0.0008 (10)

### Geometric parameters (Å, °)

**Table:**

O1—C7	1.260 (2)	C8—C9	1.356 (2)
O2—C17	1.325 (2)	C8—H8	0.9300
O2—H2B	0.870 (9)	C9—C10	1.485 (2)
N1—C7	1.379 (2)	C10—H10A	0.9600
N1—N2	1.380 (2)	C10—H10B	0.9600
N1—C1	1.415 (2)	C10—H10C	0.9600
N2—C9	1.340 (2)	C11—C16	1.381 (2)
N2—H2A	0.907 (9)	C11—C12	1.382 (2)
N3—C17	1.355 (2)	C12—C13	1.383 (2)
N3—N4	1.379 (2)	C12—H12	0.9300
N3—C11	1.418 (2)	C13—C14	1.369 (3)
N4—C19	1.323 (2)	C13—H13	0.9300
C1—C6	1.381 (2)	C14—C15	1.365 (3)
C1—C2	1.381 (2)	C14—H14	0.9300
C2—C3	1.378 (2)	C15—C16	1.384 (2)
C2—H2	0.9300	C15—H15	0.9300
C3—C4	1.366 (3)	C16—H16	0.9300
C3—H3	0.9300	C17—C18	1.366 (2)
C4—C5	1.362 (3)	C18—C19	1.392 (2)
C4—H4	0.9300	C18—H18	0.9300
C5—C6	1.383 (2)	C19—C20	1.498 (2)
C5—H5	0.9300	C20—H20A	0.9600
C6—H6	0.9300	C20—H20B	0.9600
C7—C8	1.400 (2)	C20—H20C	0.9600
			
C17—O2—H2B	109.5 (13)	C9—C10—H10B	109.5
C7—N1—N2	108.62 (11)	H10A—C10—H10B	109.5
C7—N1—C1	129.74 (12)	C9—C10—H10C	109.5
N2—N1—C1	120.42 (11)	H10A—C10—H10C	109.5
C9—N2—N1	107.91 (11)	H10B—C10—H10C	109.5
C9—N2—H2A	124.3 (11)	C16—C11—C12	120.50 (14)
N1—N2—H2A	120.4 (12)	C16—C11—N3	118.91 (13)
C17—N3—N4	110.49 (11)	C12—C11—N3	120.57 (13)
C17—N3—C11	129.60 (11)	C11—C12—C13	118.88 (17)
N4—N3—C11	119.90 (10)	C11—C12—H12	120.6
C19—N4—N3	105.14 (11)	C13—C12—H12	120.6
C6—C1—C2	120.05 (14)	C14—C13—C12	121.09 (19)
C6—C1—N1	119.90 (13)	C14—C13—H13	119.5
C2—C1—N1	120.05 (13)	C12—C13—H13	119.5
C3—C2—C1	119.32 (17)	C15—C14—C13	119.52 (17)
C3—C2—H2	120.3	C15—C14—H14	120.2
C1—C2—H2	120.3	C13—C14—H14	120.2
C4—C3—C2	120.86 (18)	C14—C15—C16	120.93 (19)
C4—C3—H3	119.6	C14—C15—H15	119.5
C2—C3—H3	119.6	C16—C15—H15	119.5
C5—C4—C3	119.69 (17)	C11—C16—C15	119.06 (18)
C5—C4—H4	120.2	C11—C16—H16	120.5
C3—C4—H4	120.2	C15—C16—H16	120.5
C4—C5—C6	120.84 (18)	O2—C17—N3	119.60 (12)
C4—C5—H5	119.6	O2—C17—C18	133.19 (13)
C6—C5—H5	119.6	N3—C17—C18	107.21 (12)
C1—C6—C5	119.23 (17)	C17—C18—C19	105.80 (12)
C1—C6—H6	120.4	C17—C18—H18	127.1
C5—C6—H6	120.4	C19—C18—H18	127.1
O1—C7—N1	122.01 (13)	N4—C19—C18	111.36 (13)
O1—C7—C8	132.37 (13)	N4—C19—C20	119.87 (13)
N1—C7—C8	105.62 (12)	C18—C19—C20	128.74 (14)
C9—C8—C7	108.35 (13)	C19—C20—H20A	109.5
C9—C8—H8	125.8	C19—C20—H20B	109.5
C7—C8—H8	125.8	H20A—C20—H20B	109.5
N2—C9—C8	109.29 (14)	C19—C20—H20C	109.5
N2—C9—C10	119.47 (14)	H20A—C20—H20C	109.5
C8—C9—C10	131.23 (15)	H20B—C20—H20C	109.5
C9—C10—H10A	109.5		

### Hydrogen-bond geometry (Å, °)

**Table:**

*D*—H···*A*	*D*—H	H···*A*	*D*···*A*	*D*—H···*A*
N2—H2A···N4^i^	0.91 (1)	1.90 (1)	2.7999 (17)	169.(2)
O2—H2B···O1	0.87 (1)	1.62 (1)	2.4813 (15)	171.(2)

Symmetry codes: (i) *x*, *y*−1, *z*.

## References

[bb1] Allen, F. H., Kennard, O., Watson, D. G., Brammer, L., Orpen, A. G. & Taylor, R. (1987). *J. Chem. Soc. Perkin Trans. 2*, pp. S1–19.

[bb2] Bruker (2002). *SAINT* and *SMART* Bruker AXS Inc., Madison, Wisconsin, USA.

[bb3] Sheldrick, G. M. (1996). *SADABS* University of Göttingen, Germany.

[bb4] Sheldrick, G. M. (2008). *Acta Cryst.* A**64**, 112–122. 10.1107/S0108767307043930 18156677

[bb5] The Edaravone Acute Infarction Study Group (2003). *Cerebrovasc. Dis.* **15**, 222–229. 10.1159/000069318 12715790

[bb6] Watanabe, T., Yuki, S., Egawa, M. & Nishi, H. (1994). *J. Pharmacol. Exp. Ther.* **268**, 1597–1604. 8138971

